# Experimental Investigation of the Crystallization and Thermal Behavior of Animal Fat Mixtures Using a Multi‐Technique Approach

**DOI:** 10.1002/mnfr.70485

**Published:** 2026-05-01

**Authors:** Michele Lessona, Antoine Cros, Laurent Sagalowicz, Cynthia Marmet, Antonio Buffo, Elena Simone

**Affiliations:** ^1^ Department of Applied Science and Technology (DISAT) Politecnico di Torino Torino Italy; ^2^ Nestlé Research Vers‐chez‐les‐Blanc Lausanne Switzerland

**Keywords:** animal fats, crystallization, polymorphism, trialcylglycerols

## Abstract

Fats are essential ingredients widely used in the food industry, as well as in cosmetic and pharmaceutical formulations. Solid fats are complex multicomponent systems primarily composed of triacylglycerols (TAGs), which determine the types and properties of the crystalline structures formed. TAGs crystallize in different polymorphs and stacking configurations, with distinct thermal and mechanical properties that influence the macroscopic structure and sensory profile of fat‐based products. In this study, a comprehensive multi‐technique analysis of animal‐derived fats, specifically chicken and beef fats, was conducted. Chemical characterization was performed and solid fat content (SFC) was determined. Thermal behaviour was investigated using differential scanning calorimetry (DSC), whereas crystallization experiments were conducted using in situ turbidity measurements and synchrotron small‐angle and wide‐angle x‐ray scattering (SAXS/WAXS) for structural characterization. Three different synchrotron experimental setups were used for crystallization experiments, including static and sheared conditions. The results demonstrate that the crystallization behaviour of beef and chicken fat samples closely correlate with their TAGs composition. Synchrotron x‐ray scattering provided structural insights, highlighting how the polymorphic behaviour is influenced by fat origin and crystallization conditions. For both animal fat types, all three main polymorphs and possible transitions were detected. Moreover, the presence of shear promoted crystallization of stable polymorphs.

## Graphical abstract

A multi‐technique analysis of animal‐derived fats, specifically chicken and beef fats was conducted. Chemical characterization was performed and solid fat content was determined. Thermal behavior was investigated using differential scanning calorimetry, crystallization experiments were conducted using in situ turbidity measurements and synchrotron small‐angle and wide‐angle X‐ray scattering for structural characterization.

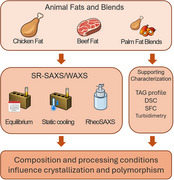

## 1 Introduction

Fats and oils are one of the three main human macronutrients. They are fundamental to the human diet due to their high energy content and the ability to solubilize essential micronutrients, such as lipophilic vitamins A, D, and E [1]. For these reasons, fats and oils are widespread in‐home cooking and for industrial food preparation. In particular, solid fats are key ingredients in food, providing the desired texture, and palatability of the final product. Aside from the food industry, fats are also widespread ingredients in cosmetic and pharmaceutical formulations.

Natural edible fats are mainly constituted by a mixture of several triacylglycerols (TAGs). TAGs are composed of a glycerol molecule attached to three fatty acid (FA) molecules. The chemical complexity of edible fat mixtures comes from the fact that many different FAs exist, with differences in terms of their level of saturation and the length of the acyl chains. Moreover, it is also important to consider the number of different FAs in the same TAG molecule and their position, as these two parameters can influence the solid‐state behavior [2, 3].

TAG molecules crystallize in different crystal structures (polymorphs). TAG crystals present three main polymorphic forms: alpha (*α*), beta prime (*β*′), and beta (*β*) reported in order of increasing melting temperature, density and overall thermodynamic stability. TAG polymorphs are monotropic, meaning that polymorphic transitions are not reversible and can only occur from the less stable *α* form to the more stable *β*′ and *β* polymorphs. TAGs polymorphs are distinguished based on the different subcells formed by the lateral FA chain packing [2, 4].

Moreover, TAG molecules crystallize with different stacking motifs, forming lamellar structures with different long‐range d‐spacing. The nomenclature for different lamellar structures is based on the number of FA chains of generic length *L* between the two ends of the lamella.

Due to the differences in molecular structure of the TAGs forming edible fat mixtures, these mixtures can exhibit a complex phase behavior, forming solid solutions, eutectic mixtures and molecular compounds, due to the different relative miscibility of their TAGs. In fact, not all TAGs molecules are equally miscible, and their miscibility heavily depends on the polymorphic form in which they crystallize [4, 5].

TAG crystallization behavior influences several important properties of fats. Different polymorphs present different melting points; tailoring polymorphism and polymorphic crystallization kinetics can have a direct influence on macroscopic properties of fat‐based products, such as mouthfeel, spread ability, texture, and melting profile. These properties in turn determine the sensorial experience of food and affect digestibility. Obtaining the desired properties of the final product requires fine‐tuning of the TAG composition and the crystallization conditions. Despite the importance of fat‐based products, the effect of TAG composition on the crystallization behavior of edible is not always clear. For this reason, a better understanding of how the composition of a fat mixture influences crystallization is needed. This is of key importance for practical industrial applications, tailoring process design to achieve the desired fat crystalline form and thus the correct product properties

Natural edible fats and oils can be extracted from many fat‐rich foods: nuts, legumes, seeds, meat, and dairy [6]. The FAs composition, and consequently, the TAG profile, differs depending on several parameters, including the source (animal or plant), the species (e.g. cow or buffalo milk fat [7], and the geographical origin or feeding [8, 9].

The global demand for oils and fats is rapidly increasing. Between 1995 and 2011, edible plant‐based oil production grew by 48% [10]. This increasing trend presents significant challenges for production and the food industry, which must consider environmental, geopolitical, socioeconomic, and cultural factors that directly influence the demand, availability and consequently the price of natural fats and oils [10, 11].

In this context, the food industry has to develop strategies to replace fats and oils originating from conventional sources with more sustainable and convenient ones. Reformulating fat‐based food products, however, is not a straightforward process as even small differences in TAG profile could cause a dramatic effect on the texture, stability, and mouthfeel properties of the final product [12].

Over the years, significant efforts have been made to reduce and/or replace conventional fats and oils. For example, the confectionery industry has been consistently looking for ways to replace cocoa butter by using blends of other plant‐based fats [12, 13, 14]. Regarding animal fat substitution, efforts have also been made to find ways to effectively replace milk fat in chocolate products [15].

Aside from dairy, other animal fats include tallow (beef fat), lard (pork fat), and chicken fat. These types of fats are mainly used for shortenings, margarines, and savory baking products due to their flavor, stability, and physical properties (e.g., solid fat content) [16, 17, 18]. This poses a challenge for food manufacturers as replacing fats of animal origin is not straightforward. The TAG composition of these fats differs substantially from that of plant‐based fats; this affects the crystallization behavior and hence, structural, thermal, and sensory properties. Without a clear understanding of the crystallization behavior of animal fats it would be extremely difficult to recreate the same macroscopic properties in vegetarian or vegan products. From a structural perspective, studies on animal tissue fats remain limited.

Preliminary studies on animal fat mixtures do exist. Faridah and coworkers [18] studied the melting behavior of chicken fat/palm oil blends with differential scanning calorimetry (DSC). Mao and coworkers [16] studied beef tallow and chicken fat with Powder x‐Ray diffraction and polarized light microscopy (PLM) showing the effect of different compositions on the crystallized blend. While these techniques provide valuable information on thermal events and microstructure, they cannot unambiguously characterize polymorphic events. Bench‐top methods also have limited ability to follow structural evolution in real time during non‐equilibrium ramps. By contrast, time‐resolved synchrotron SAXS/WAXS tracks lamellar structure and polymorph transitions in situ during controlled thermal protocols.

In this work, a multi‐technique approach to the investigation of animal fat crystallization is reported. In particular, chicken and beef fat samples were first characterized from a chemical point of view (FA and TAG composition); then their crystallization behavior was investigated using bench‐top techniques (turbidimetry and DSC) and, time‐resolved synchrotron radiation x‐ray scattering in the Small‐ and Wide‐Angle regions (SAXS/WAXS). The synchrotron experiments were performed using three different setups, including both static and shear conditions (RheoSAXS), enabling direct tracking of lamellar structure development (SAXS) and polymorphic evolution (WAXS) during controlled cooling and heating ramps. This approach provides a deeper structural understanding of the crystallization behavior of animal tissue fats, highlighting the effect of different processing conditions.

## 2 Methodology

### 2.1 Materials

Chicken fat samples, beef fat samples, and their blends with palm fats and oleic acid were prepared. CF1 and CF2 are chicken fat fractions, CF3 is a blend of CF2 and 10% by weight of palm fat. BF1 is Beef Fat and BF2 is a blend of BF1 and 10% by weight of oleic acid. The 10% (w/w) addition levels were selected as a modification relevant to food formulation. This addition produces macroscopically measurable changes in crystallization and melting behavior while maintaining the animal fat as the predominant component of the blend.

### 2.2 Chemical Characterization

The TAG profile was obtained through a mass spectrometry approach. This approach consists of a Non‐Aqueous Reversed Phase Liquid Chromatography (NARP‐LC) coupled with an ElectroSpray Ionization Mass Spectrometry (ESI‐MS). This setup enables the regiospecific analysis of TAGs, allowing a correct determination of the TAG profile. The detailed procedure is reported in previous work [19].

### 2.3 Solid Fat Content (SFC)

Solid fat content (SFC) curves were collected by pulsed Nuclear Magnetic Resonance (p‐NMR) following the ISO 8292‐:2008 standard methodology for animal and vegetable fats and oils [20].

The temperatures at which the SFC values were collected were different among samples, due to their different melting ranges. For samples CF1, CF2 and BF1, the SFC values were measured every 5°C, while the SFC of samples CF3 and BF2 was measured every 10°C with the exception of the interval between 30°C and 40°C, corresponding to mouth temperature which is of greater interest for food applications, where measurements were acquired every 5°C.

### 2.4 Differential Scanning Calorimetry DSC

Heat flow measurements were performed using a Hitachi DSC200 thermal analysis system (Hitachi, Japan). An approximate amount of 3 mg of each sample was weighed and sealed in an aluminium pan, while an identical empty pan was used as a reference. Each sample was heated up to at 80°C and then held at that temperature for 5 min. Samples were cooled to 15°C at −5°C/min and then kept at that temperature for 10 min before heating up to 80°C with a heating rate of 5°C/min.

### 2.5 Synchrotron small‐angle X‐ray Scattering—Capillary Holder

Samples at equilibrium (i.e. after 8 months of storage at ambient conditions) were analyzed at the SAXS beamline at Elettra Sincrotrone Trieste (Italy). A capillary holder with temperature‐controlled setup was used. Static measurements at 20°C were performed. The samples were prepared 8 months before measurement by melting in an oven at 70°C, then filling quartz capillaries with diameter of 1.5 mm with each sample. The SAXS scattering patterns were collected using a Pilatus3 1 M detector (Dectris DH) was used to detect the SAXS signal (*range* = 0.761/Å *< q <* 57.5 1/Å) while a Pilatus 100k detector was used for the analysis of the WAXS region (*range* = 63 1/Å *< q <* 163 1/Å); the beam energy was 8 keV(1.54 Å wavelength).

### 2.6 Synchrotron small‐angle X‐ray Scattering—Multi‐capillary Holder

The polymorphs arising from crystallization of each sample were determined using synchrotron radiation X‐ray scattering (*λ* = 0.69 Å) at the I22 beamline of Diamond Light Source (DLS, Didcot,U.K.).

At DLS, a Pilatus P3‐2 M from Dectris was used as the SAXS detector, whereas for WAXS a Pilatus3‐2M‐DLS‐L detector was installed. The beam energy used at I22 was 18 keV. For all samples and synchrotron setups, calibration was performed with silver behenate (d‐spacing 58.38 Å) for the SAXS region and p‐bromobenzoic acid for the WAXS region. A quartz capillary with a diameter of 1.7 mm and a length of approximately 80 mm was used as the sample holder. An empty capillary of the same size was used as the reference.

The temperature was initially set to 70°C for 10 min, and then decreased to 20°C at a rate of ‐1°C/min; the samples were then kept at that temperature for roughly 2 h and then heated up again at a rate of 1°C/min until fully melted. The exposure time was set to 1.0 s. Diffraction images were processed using the software DAWN [21]. The resulting data were extracted, sorted and analyzed using Python, Excel 2023 and Origin Pro 2018 (peak fitting function).

### 2.7 Synchrotron small‐angle X‐ray Scattering—Rheometer

Another set of SAXS/WAXS experiments was performed at beamline ID02 at the European Synchrotron (ESRF) located in Grenoble (France), using the stress‐controlled rheometer setup (a commercial Thermo‐Haake RS6000) equipped with an X‐ray transparent Couette cell. The concentric cylinders of the cell used (inner diameter 20 mm, outer diameter 22 mm, and height 40 mm) were machined out of Vespel polymer. An Eiger24M detector (Dectris, DH) was used for the SAXS signal (0.45*< q <* 58.4 1/Å), while a Rayonix LX 170HS (Rayonix, US) detector was used for the WAXS (56.8*< q <* 351.7 1/Å). The energy of the beam was 12.5 keV (1 Å wavelength). Analyzed samples were melted at 70°C in an oven and then around 2 mL of sample was transferred into the Couette cell using a pipette. The temperature was raised to 70°C and then cooled down to 20°C at a cooling rate of −1°C/min. The samples were then kept at the same temperature for 2 h and then reheated again at a rate of 1°C/min until fully melted. During the cooling and the heating, the samples were subjected to a shear of 1000/s, in line with previous similar works [12, 22].

### 2.8 Turbidity

Turbidity measurements were performed using a Technobis CrystalBreeder platform (Technobis, NL). Roughly 1 mL of the different samples was inserted into glass vials, which were then placed in controlled‐temperature reactors. A 645 nm light source was used for the turbidity detection. All samples were fully melted at 70°C and kept for 15 min to erase crystal memory, at this point the transmissivity was set to 100% as a baseline. A cooling programme of −1°C/min was applied and the samples were cooled to 15°C and kept for 150 min. Samples were then heated with to 70°C at 1°C/min. Throughout the procedure, transmissivity data were collected every 1 0s.

## 3 Results and Discussion

### 3.1 Chemical Composition

All the investigated fat samples exhibited a complex TAGs profile, with more than 50 different TAG species present. The simplified TAG and FAs profiles are reported in Table 1.

**TABLE 1:** Table containing the FA and the TAG profiles of the samples, expressed as 1g/100g. *Limit of quantification 0.2 g/100 g.

	Sample	CF1	CF2	CF3	BF1	BF2
TAG	Tri‐Saturated Mono‐Unsaturated Symmetrical	*<*1 18.4 8.2	1.6 19.8 2.8	4.5 22.6 3.3	7.4 39.2 11.2	7.4 39.2 11.2
	Asymmetrical	10.2	17.0	19.4	28.1	28.0
	Di‐Unsaturated	41.9	42.1	39.9	46.1	46.11
	Tri‐Unsaturated	39.7	36.5	32.9	7.3	7.3
	TAG species*	57	58	64	60	60
FA	Chain Length Parity	Even	Even	Even	Even, Odd	Even, Odd
	Saturated	29.2	27.2	31.2	50.9	45.1
	Monounsaturated	54.2	54.2	51.5	47.3	53.3
	PUFA	16.7	18.6	17.3	1.7	1.5
	Mean Chain Length	17.4	17.4	16.7	17.2	17.3

An immediate distinction between chicken fat samples and beef fat samples in terms of their TAG composition can be made: the content of tri‐unsaturated TAGs (i.e. TAGs for which all three chains contain at least one unsaturated bond) is higher in samples CF1, CF2, and CF3 than in beef fat‐based samples. The inverse trend can be observed for mono‐unsaturated TAGs, whose content is higher in BF1 and BF2 than in the other samples.

It is also interesting to look at the FA profiles. It is possible to notice the presence of FAs with an odd‐carbon‐ number of carbon atoms in samples BF1 and BF2. In general, the vast majority of fats and oils of natural origin are composed of FAs with an even number of carbon atoms; however, fats of ruminant origin (such as beef fat) are reported to contain both even‐ and odd‐carbon‐number fatty acid chains due to their characteristic ruminant microbiome [23]. Samples of chicken origin have a higher content of polyunsaturated fatty acids (PUFAs) compared with beef fats. The average number of carbon atoms in all FAs for each sample is rather similar.

### 3.2 Solid Fat Content Curve

**FIGURE 1:**
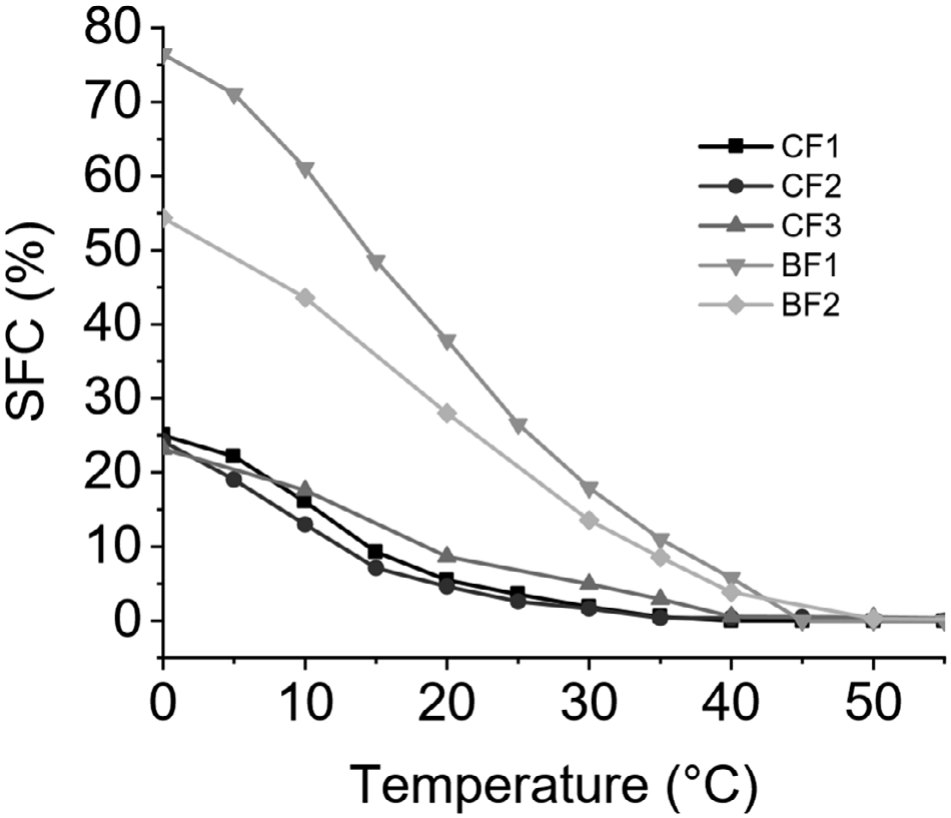
Solid Fat Content curves of the analysed samples. Beef fat samples show an overall higher SFC value, the addition of oleic acid and palm fat causes a reduction in SFC.

The melting behavior of the analyzed samples can be correlated with their TAG composition. The solid fat content curve gives an immediate picture of the solid percentage at a given temperature. From the SFC curves of the samples investigated (Figure 1) it can be seen that, in general, chicken fat samples (CF1, CF2 and CF3) have a lower solid fat content than the beef fat samples (BF1 and BF2). Compared with BF1, BF2 shows a reduced SFC due to the addition of oleic acid, especially at lower temperatures.

In general, the SFC curves, and consequently the melting profile, are well correlated with the samples’ composition; higher‐melting samples are richer in saturated and monounsaturated FAs than the lower melting samples which are richer in polyunsaturated FAs as reported in Section 3.1.

Blending animal fat with plant‐based fats or oleic acid (samples CF3 and BF2, respectively) influences the melting behavior of the unaltered animal fat samples (CF2 and BF1, respectively). In particular, the effect of the addition of 10% palm fat causes a slight increase in the SFC of CF3 compared with CF2 between 10°C and 40°C, while the addition of 10% oleic acid to BF1 has a more pronounced impact on the SFC of BF2 at lower temperatures.

### 3.3 X‐ray Scattering

The number and type of polymorphs that each sample could form during cooling crystallization were investigated with synchrotron x‐ray scattering. This technique enables fast and high‐resolution measurements, which are able to capture the rapid phase transitions that may occur in crystallizing fat samples. The lamellar long d‐spacing was obtained from the SAXS patterns by taking the first order reflection q values and calculating the respective short spacing through the following equation:

(1) d=2π2πqq

If higher‐order reflections were found, the linear interpolation method described in Pratama et al. [24], was used. This interpolation method also allowed the distinction of which peak belonged to which phase in the case of multiphase systems.

The samples polymorphism was addressed by analysing the WAXS region. The main polymorphs (*α*, *β*′, and *β*) were identified thanks to characteristic peaks commonly associated with a specific polymorph [25, 26, 27]. The distinctive peaks of the three main polymorphs are reported in Table 2.

**TABLE 2:** Characteristic q values and short spacing linked to TAG polymorphs, values collected from [25].

Polymorph	q vector (nm^−1^)	Short spacing (nm)
*α*	15.14	0.415
*β*′	14.96 and 16.53 or 14.71, 15.83 and 16.93	0.42 and 0.38 or 0.427, 0.397 and 0.371
*β*	13.66	0.46

The following section will report the patterns obtained in three different setups: first the equilibrium condition of the samples was investigated in a static experiment at fixed temperature; in these measurements, WAXS reflections are typically well developed and less affected by the liquid background, allowing the most confident polymorphic assignment. Then the crystallization behavior was investigated in a cooling experiment in static conditions and finally during cooling under shear in order to investigate the crystallization conditions in a dynamic setup. In the time‐resolved cooling experiments, polymorphic assignments can be more tentative at early times and low solid fat content (SFC) due to weaker and/or overlapping WAXS peaks; therefore, for low‐SFC samples, precise polymorphic attribution is in some cases made by comparing the behavior to similar samples showing clearer reflections.

#### 3.3.1 Equilibrium Capillary Setup

**FIGURE 2:**
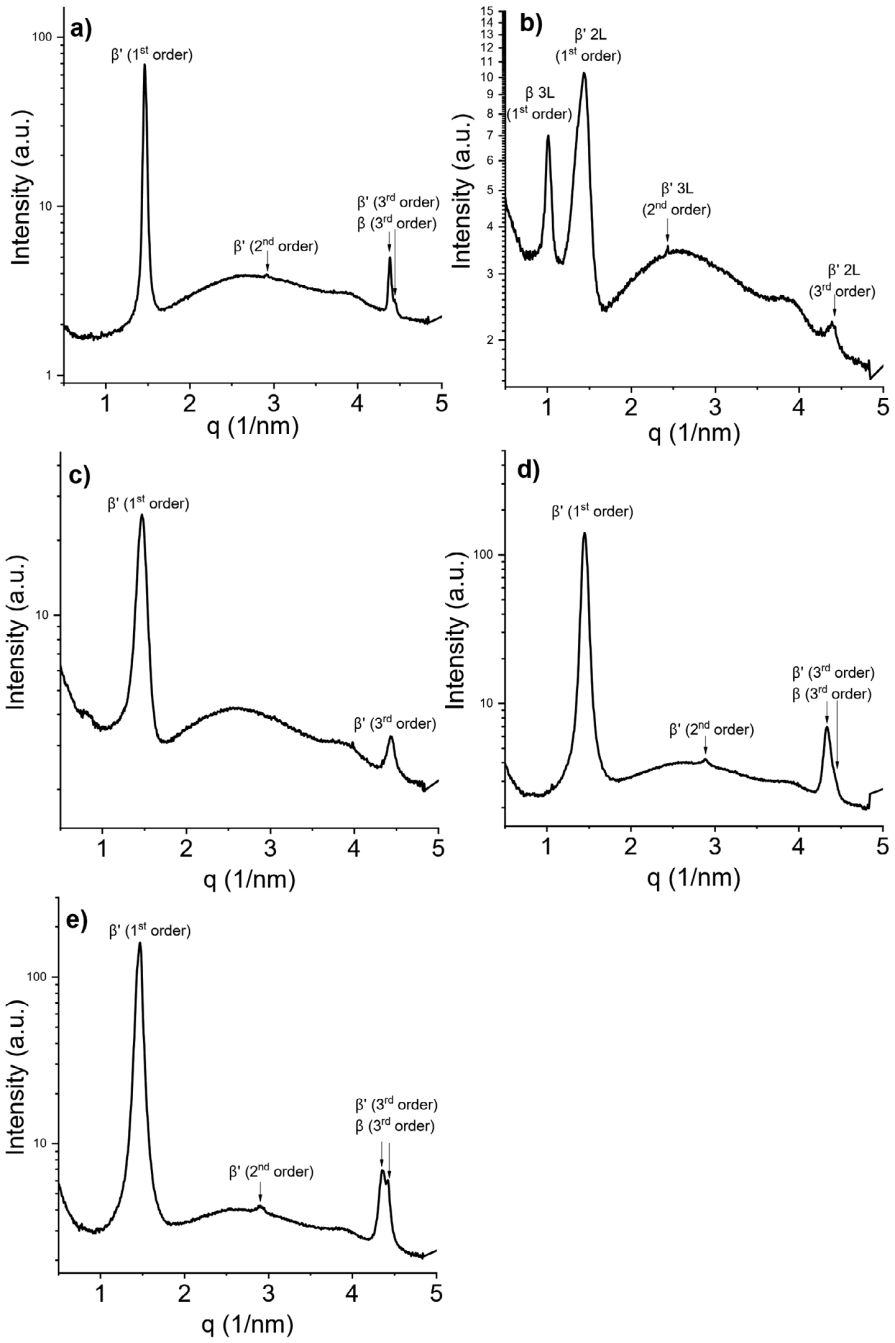
SAXS diffractograms of: (a) CF1, (b) CF2, (c) CF3, (d) BF1, and (e) BF2 acquired at 20°C after 8 months of storage, showing the different phases and lamellar stacking of the samples at the equilibrium.

**FIGURE 3:**
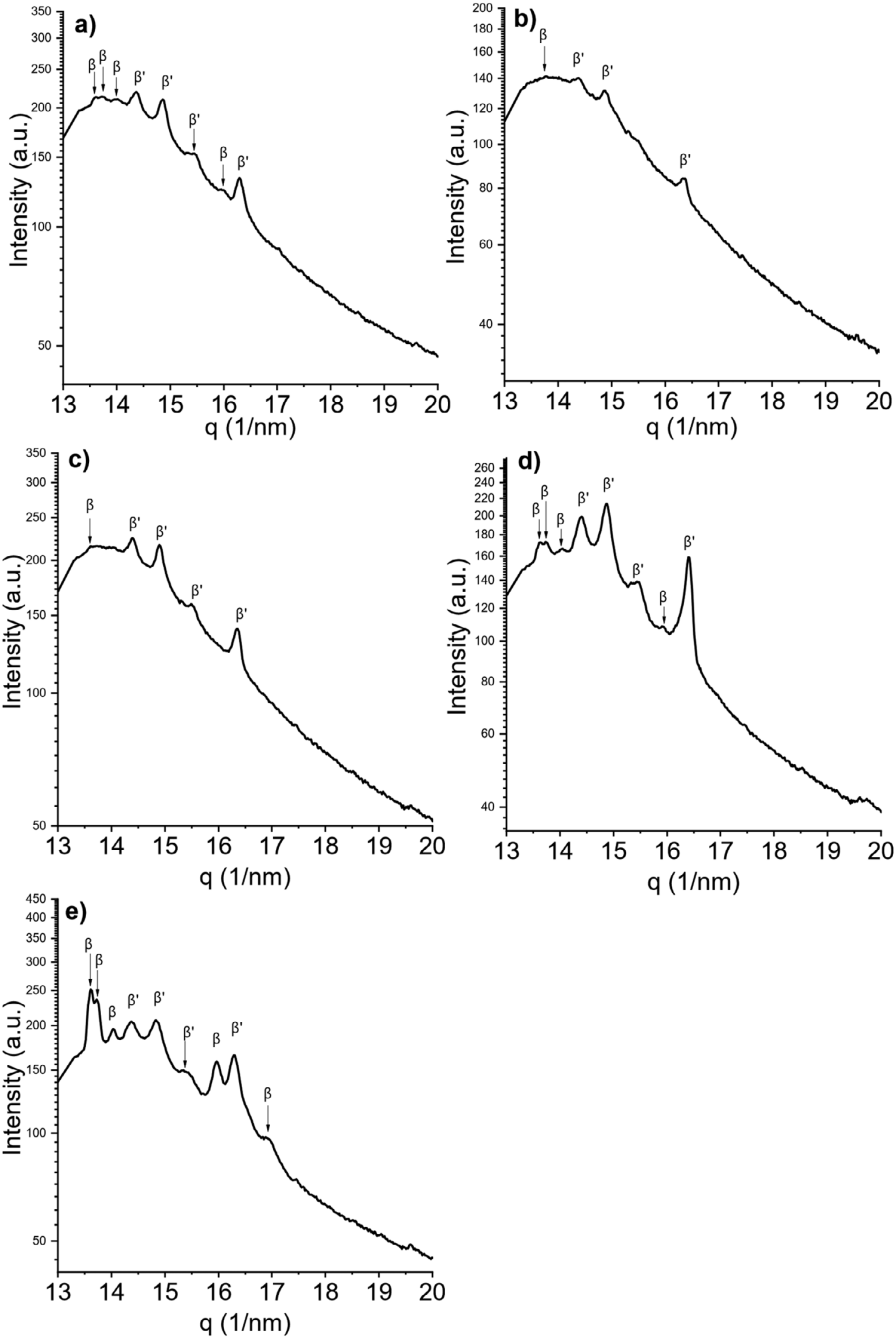
WAXS diffractograms of: (a) CF1, (b) CF2, (c) CF3, (d) BF1, and (e) BF2 acquired at 20°C after 8 months of storage, showing the polymorphic behavior of the samples at the equilibrium.

The equilibrium behavior was investigated by measuring the samples after 8 months of room temperature storage. The SAXS pattern of CF1 shows two coexisting 2*L* lamellar phases with similar long spacings. This is evident from the presence of two partially overlapping third‐order peaks, with a maximum at 4.38nm^−1^ and a weaker peak at slightly higher *q* values. In the first‐order region, only a single broad peak is observed, so the contributions of the two phases cannot be clearly distinguished (Figure 2a). The long spacing of the dominant phase is 4.29 nm while the minor phase d‐spacing can be estimated from the third‐order reflection to be 4.24 nm. Polymorphs were assigned from the WAXS pattern (Figure 3a), which indicates small amounts of the *β* form via its characteristic reflection at 13.6nm−^1^. A nearby peak at 13.73nm−^1^ can also be attributed to the *β* polymorph phase [28], a weaker peak at 14.02nm^−1^ is detected only in presence of *β* crystals and was attributed to that polymorph as well. Stronger reflections at 14.86nm^−1^ and 16.29nm^−1^ are characteristic of *β*′. The remaining strong reflection at 14.39nm^−1^ has been attributed to a *β*′ polymorph [29] and was detected in mixed *β*′ + *β* systems [30].

The polymorphic behavior of sample CF2 is revealed to be mainly *β*′ due to the presence of WAXS peaks at 14.87nm^−1^, 16.35nm^−1^ and, additionally and similarly to the previous sample, at 14.39nm^−1^ (Figure 3b). Small peaks at around 13.6nm^−1^ also indicate the presence of a *β* phase. Two coexisting lamellar stackings are detected in the small‐angle region, showing a first‐order reflection and a second‐order reflection of a 3*L* stacking phase, at q values, respectively of 1.01 nm^−1^ and 2.44 nm^−1^, corresponding to a long spacing of 5.13 nm. The first‐ and third‐order reflections of the 2L phase are visible at 1.42nm^−1^ and 4.40nm^−1^, with a long d‐spacing of 4.42 nm (Figure 2b).

The wide‐angle pattern of sample CF3 shows *β*′ peaks comparable to those of the previous samples: the two peaks at 14.87nm^−1^ and 16.35nm^−1^ and another *β*′ peak at 14.39nm−^1^. By carefully analyzing the wide‐angle region in Figure 3c, it is also possible to notice a faint peak at 13.60nm^−1^, which is indicative of the presence of small amounts of a *β* polymorph. The SAXS region shows the presence of a single 2*L* phase with a long spacing of 4.24 nm whose first‐ and third‐order peaks are visible at q values of 1.45 nm^−1^ and 4.43 nm^−1^, respectively.The presence of small amounts of *β* can only be assumed from the WAXS peak positions. (Figure 2c).

Both beef fat samples (BF1 and BF2) display similar polymorphic behavior. The SAXS patterns (Figure 2d, e) reveal two coexisting lamellar phases in each sample. This is evident from the presence of two partially overlapping peaks in the third‐order diffraction region, one corresponding to the third‐order reflection of a lamellar phase with a larger long spacing and the other to a phase with a slightly smaller long spacing. In both samples, the first‐order reflection appears as a single broad peak, so the individual contributions of the two phases cannot be resolved, and the second‐order reflection is too weak for a detailed analysis. From the positions of the third‐order peaks, the long spacings detected in BF1 are 4.35 nm and 4.26 nm, whereas for BF2 they are 4.34 nm and 4.26 nm. Polymorphic assignment and relative abundance of the different phases can be obtained by the WAXS patterns: both BF1 and BF2 show a series of *β* peaks: the one at 13.60nm^−1^, and two other at 13.74nm^−1^ and 14.03nm^−1^. Peaks at 14.84nm^−1^ and 16.30nm^−1^ confirm the presence of a *β*′ polymorph, and it is, again, possible to detect an additional *β* peak at 14.03nm^−1^. Compared to BF1, sample BF2 presents more intense *β* peaks compared to the *β*′, plus an additional *β* shoulder at 19.60 nm.

In general, the predominant polymorph detected in all samples is *β*′; this is consistent with the high amount of unsaturated TAGs. Many samples also showed small, variable amounts of *β* phases, all of them having in common a relatively high content of saturated TAGs and/or a considerable amount of symmetric monounsaturated TAGs, which are known to pack closer together and to form *β*(3L) phases more easily than asymmetric TAGs [2, 31, 32, 33, 34]. A summary of the polymorphs and the long d‐spacings of the detected phases for all the investigated samples is summarized in Table 3.

**TABLE 3:** Polymorphs and d‐spacings of all the samples measured at the equilibrium.

Sample	Setup	Phase 1 d sp.(nm), Polym.	Phase 2 d sp.(nm), Polym.
CF1	Capillary	4.29*β* ^′^(2*L*)	4.24*β*(2*L*)
CF2	Capillary	4.42*β* ^′^(2*L*)	5.13*β*(3*L*)
CF3	Capillary	4.24*β* ^′^(2*L*)	ND*β*(2*L*)
BF1	Capillary	4.35*β* ^′^(2*L*)	4.26*β*(2*L*)
BF2	Capillary	4.34*β* ^′^(2*L*)	4.26*β*(2*L*)

#### 3.3.2 Multicapillary Setup Crystallization

**FIGURE 4:**
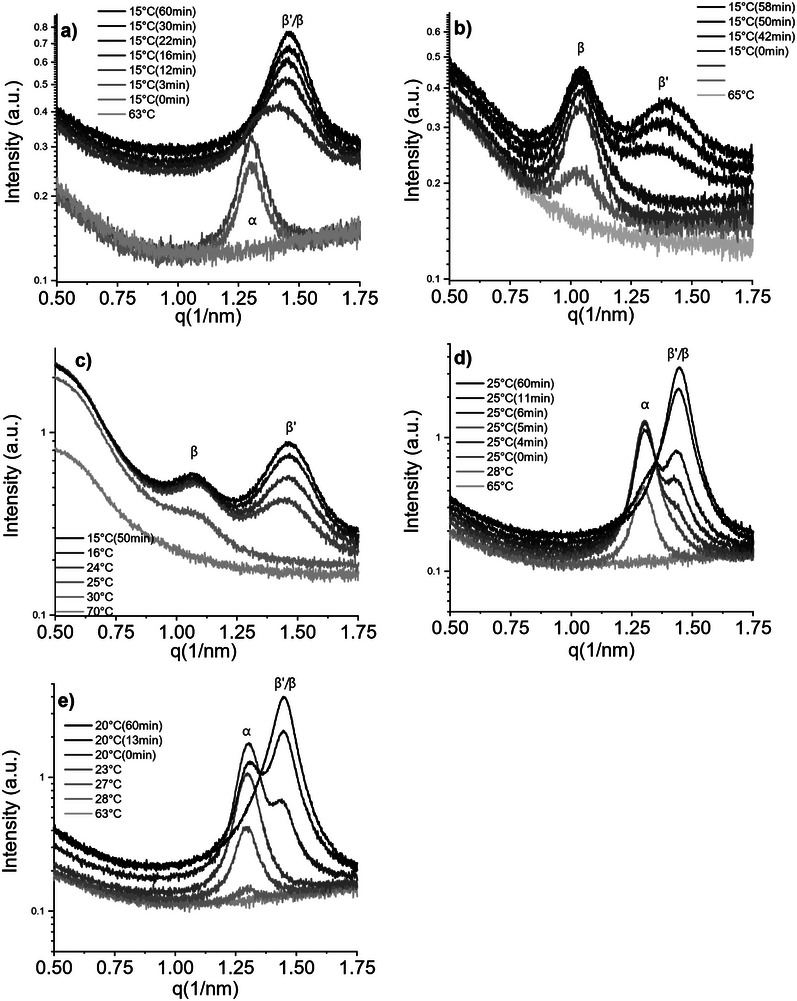
SAXS diffractograms of: (a) CF1, (b) CF2, (c) CF3, (d) BF1, and (e) BF2 acquired during cooling profiles and isothermal hold in the capillary setup, showing the different phases and lamellar stacking of the samples in static conditions as well as polymorphic transitions for samples CF1, BF1 and BF2.

**FIGURE 5:**
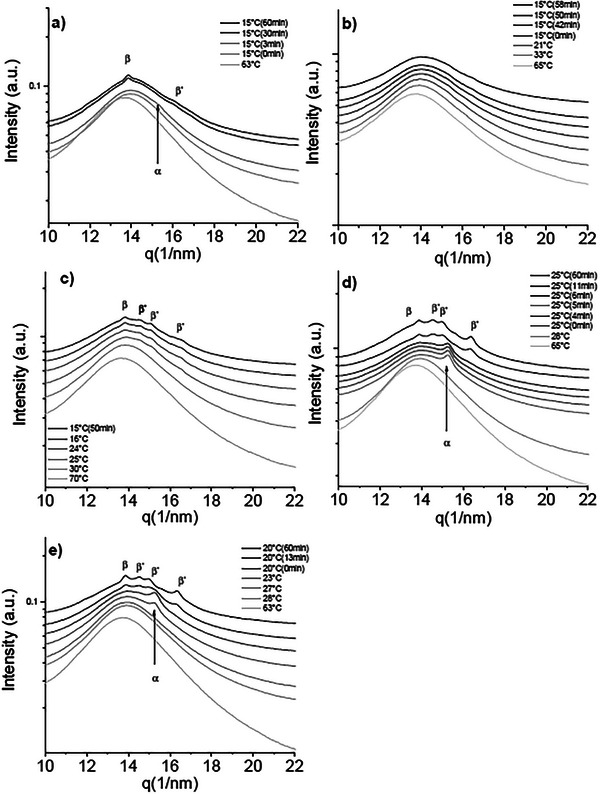
WAXS diffractograms of: (a) CF1, (b) CF2, (c) CF3, (d) BF1, and (e) BF2 acquired during cooling profiles and isothermal hold in the capillary setup, showing coexistence and polymorphic transitions during crystallisation.

The melt crystallization behavior of the samples was investigated under dynamic conditions in a capillary setup during cooling ramps and isothermal holds. The small‐angle patterns of sample CF1 clearly show a phase transition after 8 minutes of isothermal hold (Figure 4a). The first peak appears at 1.3nm^−1^ corresponding to a metastable phase with a long spacing of 4.8 nm. During the isothermal hold at 15°C, this peak progressively disappears while a second broad peak at 1.5nm^−1^ appears, corresponding to a d‐spacing of 4.3 nm. Even though the simultaneously collected WAXS pattern shows a strong liquid contribution, a very faint shoulder is barely detectable at roughly 15.2nm^−1^ corresponding to an *α* polymorph (Figure 5a). During the isothermal hold it is possible to notice a more intense peak appearing at 13.85nm^−1^, which is not exactly at the position of the characteristic peak associated to a *β* polymorph but still lies in the q range 13.65nm^−1^–13.95nm^−1^ which can be attributed to a *β* lattice. Moreover, a faint shoulder is observed around q ≈ 13.85 nm^−1^. In combination with the presence of clear β reflections, this confirms the presence of the β polymorph; however, because β and β′ features overlap in this region, small amounts of β′ may also contribute. The SAXS patterns for sample CF2 show two coexisting lamellar phases that persist during cooling and the isothermal hold (Figure 4b). The first reflection to appear is at 1.05nm^−1^, consistent with a 3*L* phase (long spacing 6.0 nm). During the isothermal hold, a second reflection emerges at 1.40nm^−1^, corresponding to a 2*L* phase with a long spacing of 4.5 nm. In the wide‐angle region, no resolvable WAXS peaks were detected (Figure 5b), so polymorph identification is not possible directly. Based on the similarity with sample CF3 (reported below), it is possible to associate the 3L lamella with a β phase and the 2L lamella with a β′ phase; however, this assignment remains tentative and would require additional measurements for an unambiguous attribution. CF3 shows a SAXS pattern similar to CF2, with two lamellar phases coexisting during cooling and the isothermal hold (Figure 4c). The first reflection appears at 1.08nm^−1^ and is assigned to a 3*L* phase (long spacing 5.8 nm), followed by a second peak at 1.47nm^−1^ corresponding to a 2*L* phase (long spacing 4.2 nm). The WAXS pattern of CF3 displays several reflections, especially at lower temperatures and longer crystallization times (Figure 5c) due to its higher SFC at lower temperatures. A peak at 13.81nm^−1^ (short spacing 0.45 nm) can be assigned to a *β* polymorph, while reflections at 14.57nm^−1^, 15.10nm^−1^, and 16.56nm^−1^ are consistent with *β*′. A heating protocol confirmed the structure–polymorph correspondence: the 3*L* lamella melted last, indicating that it is the more thermodynamically stable *β* form, while the 2*L* lamella corresponds to a *β*′ polymorph. Finally, differences in crystallization temperatures and times between the two samples can be attributed to a higher content of saturated and monounsaturated TAGs in CF3 compared with CF2, facilitating the crystallization behavior of the former, despite the similarity of the SFC curves.

Beef tallow samples BF1 and BF2 show an identical lamellar structure formation during cooling and isothermal holding. The first peak to appear in both samples was detected at 1.30nm^−1^ corresponding to a long d‐spacing of 4.8 nm. During the course of the measurement this phase disappears and another one at 1.44nm^−1^ appears, signalling the formation of a more stable polymorph with a long d‐spacing of 4.3 nm, Figure 4d,e. Polymorph identification is performed through the investigation of WAXS peaks Figure 4d,e. The first to appear is a single peak at 15.30nm^−1^ that corresponds to a short d‐spacing typical of an *α* phase; then peaks corresponding to the more stable *β*′ and *β* polymorphs appear during the course of the crystallization. The peak at 13.81nm^−1^ can be linked to the formation of a *β* phase. Other *β*′ peaks appear at 14.53nm^−1^,14.98nm^−1^, and 16.37nm^−1^.

Overall, the phase behavior detected in the dynamic experiments matched the equilibrium behavior discussed in the previous section. The most striking differences between the two setups were detected in CF3. As opposed to CF2, the SAXS peak that was attributed to a 3*L* phase was not detected at the equilibrium, most likely due to the higher content in saturated TAGs that promoted the formation of 2*L* phases. To sum up, the long d‐spacing and the polymorphism of the different phases detected in the samples and their polymorphism of the samples measured at DLS are summarized in Table 4.

**TABLE 4:** Polymorphs and d‐spacings of all the samples measured during cooling and isothermal profiles with the capillary setup.

Sample	Setup	Phase1 d sp.(nm), Polym.	Phase2 d sp.(nm), Polym	Phase3 d sp.(nm), Polym
CF1	Capillary	4.8*α*	4.3*β* ^′^(2*L*)*	4.3*β*(2*L*)
CF2 CF3	Capillary Capillary	// //	4.5*β* ^′^(2*L*)∗ 4.2*β* ^′^(2*L*)	6.0*β*(3*L*)∗ 5.8*β*(3*L*)
BF1	Capillary	4.8*α*	4.3*β* ^′^(2*L*)	4.3*β*(2*L*)
BF2	Capillary	4.8*α*	4.3*β* ^′^(2*L*)	4.3*β*(2*L*)

#### 3.3.3 RheoSAXS Setup Crystallization

From a practical point of view, it is also important to investigate the crystallization behavior of a fat when external forces are applied. This was possible thanks to the RheoSAXS setup available at ESRF, described in detail in the methodology section. It is well known that the application of shear to crystallizing TAGs can deeply influence their polymorphic behavior [35, 36]. Compared to the patterns obtained in static setups, it is possible to notice different crystallization behavior in some of the samples.

**FIGURE 6:**
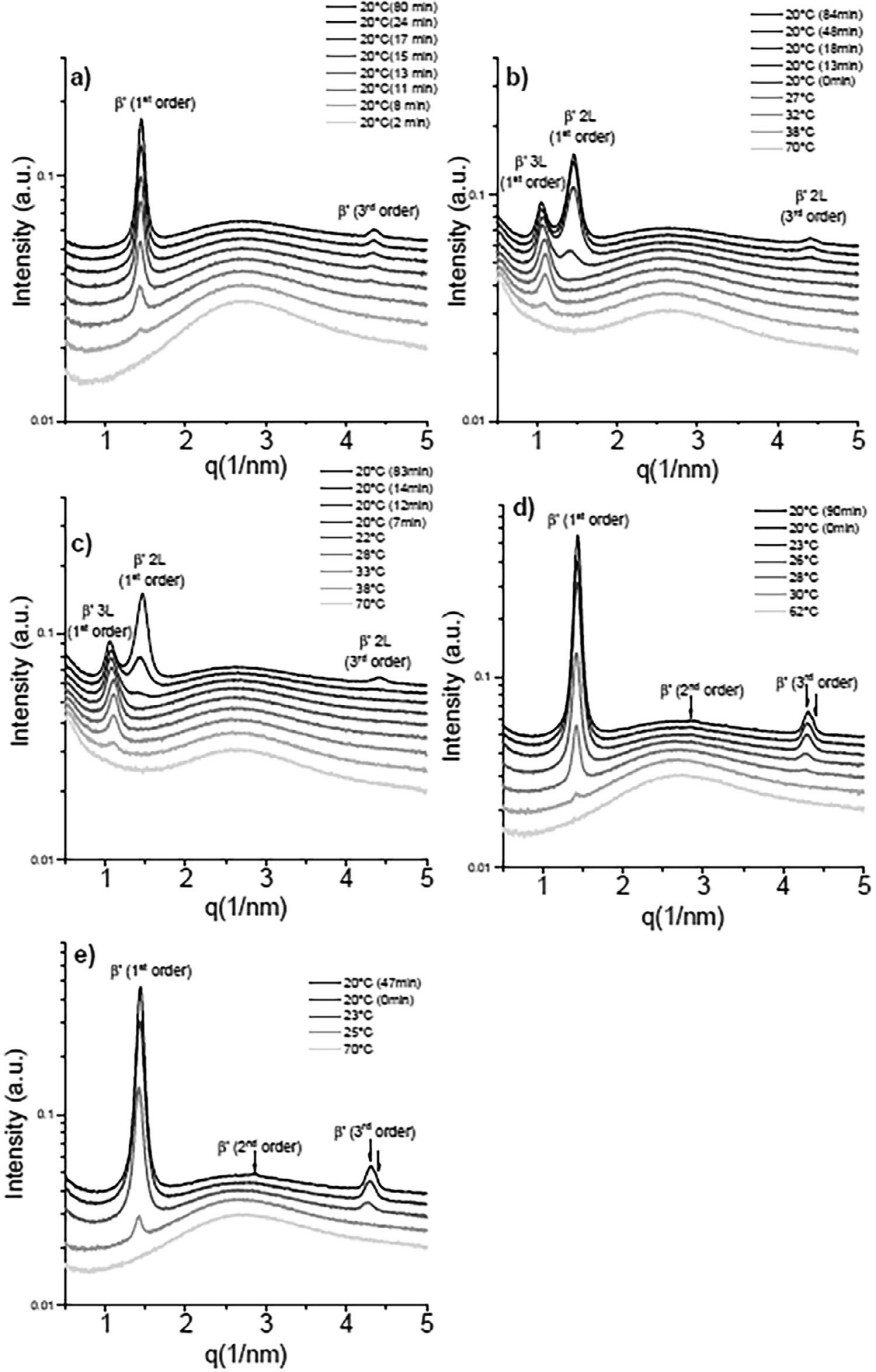
SAXS diffractograms of: (a) CF1, (b) CF2, (c) CF3, (d) BF1, and (e) BF2 acquired during cooling profiles and isothermal hold at 20°C while shear (1000 1/s) is applied. In samples CF1, BF1, and BF2 the β’ phase is formed directly.

**FIGURE 7:**
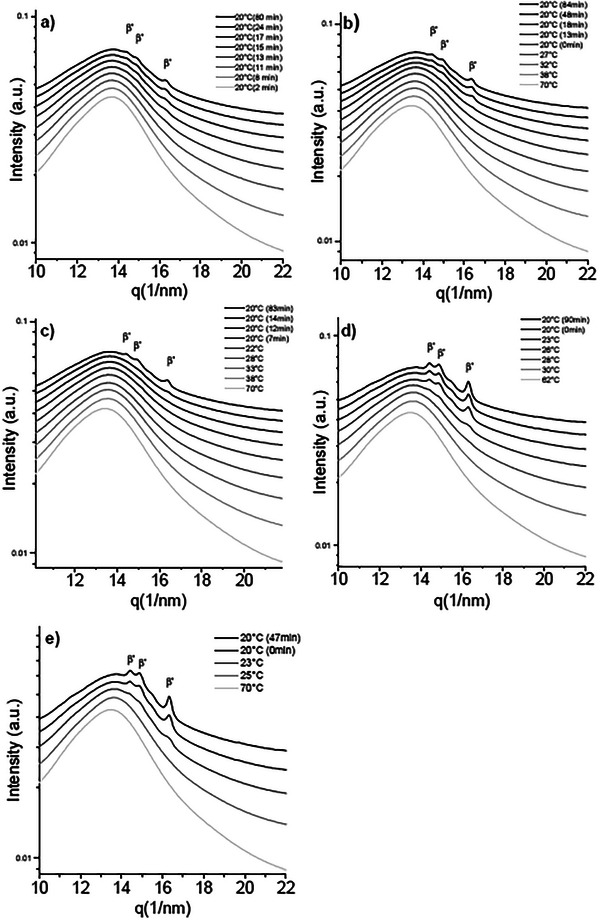
WAXS diffractograms of: (a) CF1, (b) CF2, (c) CF3, (d) BF1, and (e) BF2 acquired during cooling profiles and isothermal hold while shear (1000 1/s) is applied. In samples CF1, BF1, and BF2 the β’ phase is formed directly.

Sample CF1, compared with the static crystallization experiments, shows the formation of the *β*′ polymorph only. *β*′ peaks in the wide‐angle region (Figure 7a) are located at 14.19nm^−1^, 16.32nm^−1^, and 14.45nm^−1^. The lamellar structure produces first‐ and third‐order reflections in the SAXS region (Figure 6a) at q values of 1.44nm^−1^ and 4.35nm^−1^, respectively, which correspond to a long d‐spacing of 4.33 nm (Figure 6a).

The crystallization under shear of sample CF2 showed rather similar behavior to the static crystallization experiment. The main difference is the absence of *β* peaks in the WAXS region; in fact, the detected peaks are those at 14.97nm^−1^, 16.37nm^−1^ and 14.48nm^−1^, corresponding to a *β*′ polymorph. The small‐angle region shows two immiscible phases with slightly different d‐spacings compared with the static experiments. In particular a 3*L* phase appears at 1.06nm^−1^ with a long spacing of 5.9 nm; the 2*L* phase has a d‐spacing of 4.3 nm indicated by a first order and a third order peak at 1.46nm^−1^ and 4.40nm^−1^, respectively.

The SAXS pattern originating from sample CF3 shows the formation of two phases, a 3*L* one with a peak at 1.08nm^−1^, corresponding to a d‐spacing of 5.84 nm, and a 2*L* one that produces a first and a third order reflection at 1.45nm−^1^ and 4.41nm^−1^, respectively, resulting from a d‐spacing of 4.27 nm. Compared with CF2, the addition of palm fat promotes the formation of the *β*′ and *β* phases that occurs at a higher temperature.

Samples BF1 and BF2 have an overall higher SFC compared to the other samples, leading to a more defined WAXS pattern. For both samples, over the course of crystallization, three peaks appear at 14.45nm^−1^, 14.88nm^−1^, and 16.33nm^−1^, these peaks correspond to a *β*′ polymorph, as shown in Figure 7d,e. In the SAXS region (Figure 6d,e) a first‐order diffraction peak is observed at 1.43nm^−1^, together with a faint second‐order peak at 2.86nm^−1^. In the region of the third‐order reflection, two partially overlapping peaks are visible at 4.31nm^−1^ and 4.36nm^−1^, indicating the coexistence of two lamellar phases. The long d‐spacings of the two *β*′ phases are very similar, with values of 4.4 and 4.3 nm.

Overall, samples CF1, BF1, and BF2 in static conditions showed an *α* → *β*′ transition, while shearing experiments show the direct formation of the *β*′.

### 3.4 DSC

**FIGURE 8:**
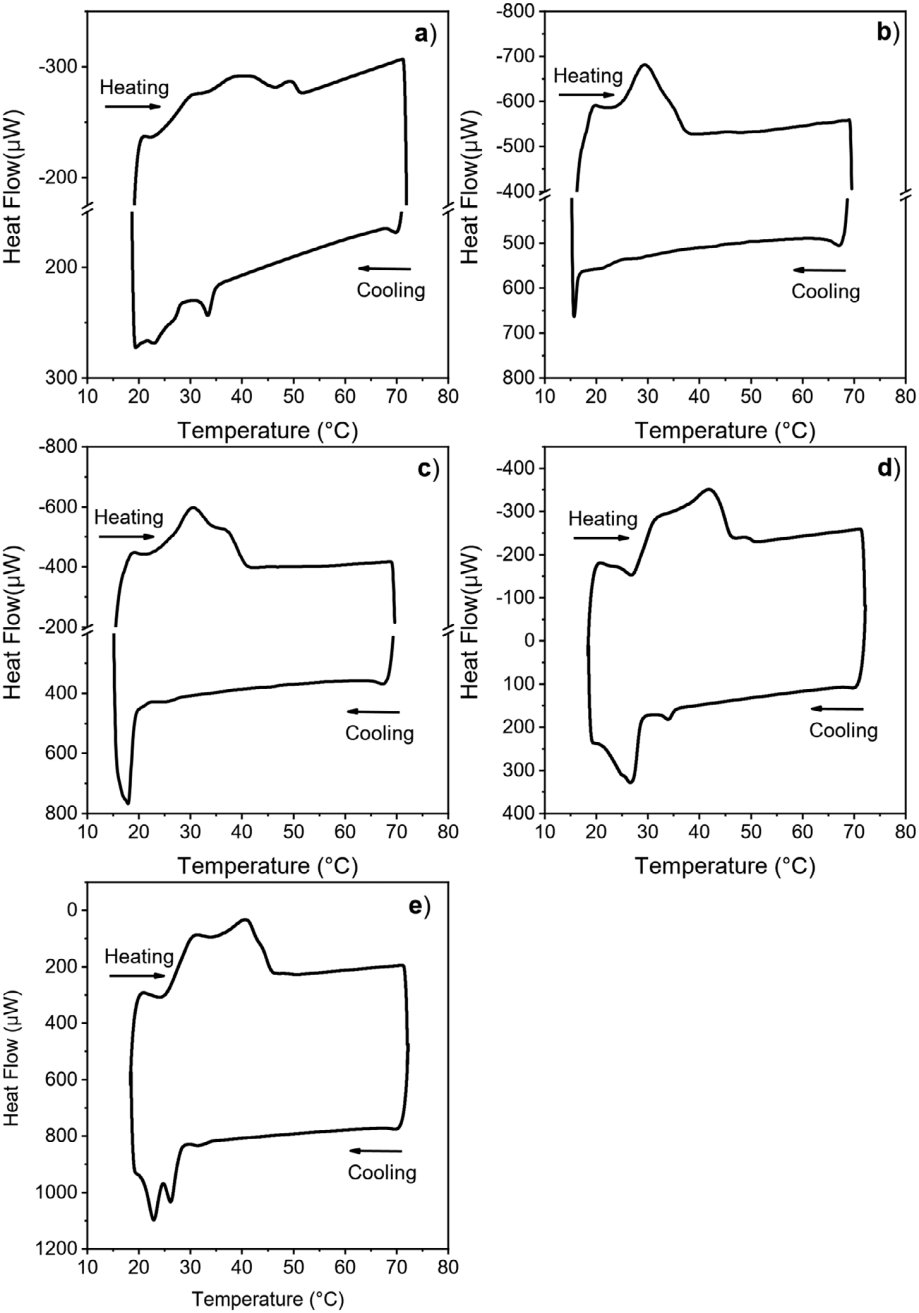
Thermograms of: (a) CF1, (b) CF2, (c) CF3, (d) BF1, and (e) BF2. ENDO UP convention is used.

Differential scanning calorimetry is a technique that is routinely employed to detect phase transitions in fat systems. Thermograms of all samples are reported in Figure 8, while a summary of all the detected transitions are reported in Table 6. DSC alone does not allow an unambiguous identification of polymorphs in complex TAG mixtures; therefore, the polymorphic attributions are based on relative polymorph stability and the SAXS/WAXS results reported in Section 3.3.

For CF1, the thermal behavior is consistent with the x‐ray results. The crystallization curve shows a clear nucleation peak that matches the formation of the α phase; at lower temperatures, three crystallization events can be detected that can be attributed to the formation of various *β*′ and *β* phases. The crystallization events at 33.4, 26.9, and 22.9°C can be attributed, based on their order, to the formation of the *α*, *β*′ and *β* polymorphs respectively (Figure 8a). Melting shows three events linked to the melting of multiple *β*′ phases and then a higher‐melting *β* phase. This higher‐melting phase can be attributed to a higher amount of symmetrical TAGs compared with the other chicken fat samples.

Sample CF2 presents two crystallization peaks, a weak one at 20.3°C and a second one at 15.6°C (Figure 8b). Based on the x‐ray results and peak order, these events are assigned to β′ and β, respectively. Heating shows two melting peaks and a shoulder corresponding to two β′ phases and a higher‐melting β one.

A similar behavior is observed for CF3 (Figure 8c), with a faint crystallization peak at 25.3°C and another at 17.8°C. The melting curve again shows three peaks, as in CF2, but with a more pronounced shoulder. This may be related to the presence of palm‐fat and the associated increase in higher‐melting saturated TAGs.

The crystallization curve of BF1 in Figure 8d shows a small peak, a larger one and a shoulder. Based on polymorphs’ thermal stability and the results from x‐ray scattering experiments it is reasonable to attribute the peak at 33.8°C to the *α* polymorph, the peak at 26.7°C to a *β*′ polymorph, and the shoulder at 24.5°C to a *β* polymorph.

Finally, BF2 (Figure 8e) presents behavior similar compared to BF1, but with more defined crystallization peaks. Crystallization peaks appear at 32.2, 26.3, and 22.8°C. Three melting events are detected, and can be assigned to the melting of the two *β*′ phases and the *β* phase, respectively. BF1 melting behavior is similar to that of BF2, aside from the different relative intensities of the highest‐melting event. This can be attributed to a higher amount of a higher amount of *β* phase than in BF1 which was detected in the scattering patterns as well.

A summary of detected melting and crystallization peaks with DSC for all samples is reported in Table 5.

**TABLE 5:** DSC curve peaks of all the analysed samples.

Sample	Heating (°C)	Cooling (°C)
CF1	30.4, 39.4, 49.7	33.4, 26.9, 22.9
CF2	19.7, 29.4, 34.9	20.3, 15.6
CF3	29.2, 30.6, 36.1	25.3, 17.8
BF1	31.7, 41.7, 48.9	33.8, 26.7, 24.5
BF2	30.9, 40.6, 44.6	32.2, 26.23, 22.8

DSC is a fast and useful technique for detecting polymorphic transitions and thermal events in general; however, without previous knowledge of x‐ray scattering experiments it would have been extremely difficult to assign which polymorph and which lamellar structure were crystallizing or melting. This is particularly true for complex TAG mixtures whose overlapping thermal peaks may make the polymorphic behavior difficult to rationalize.

### 3.5 Turbidimetry

**FIGURE 9:**
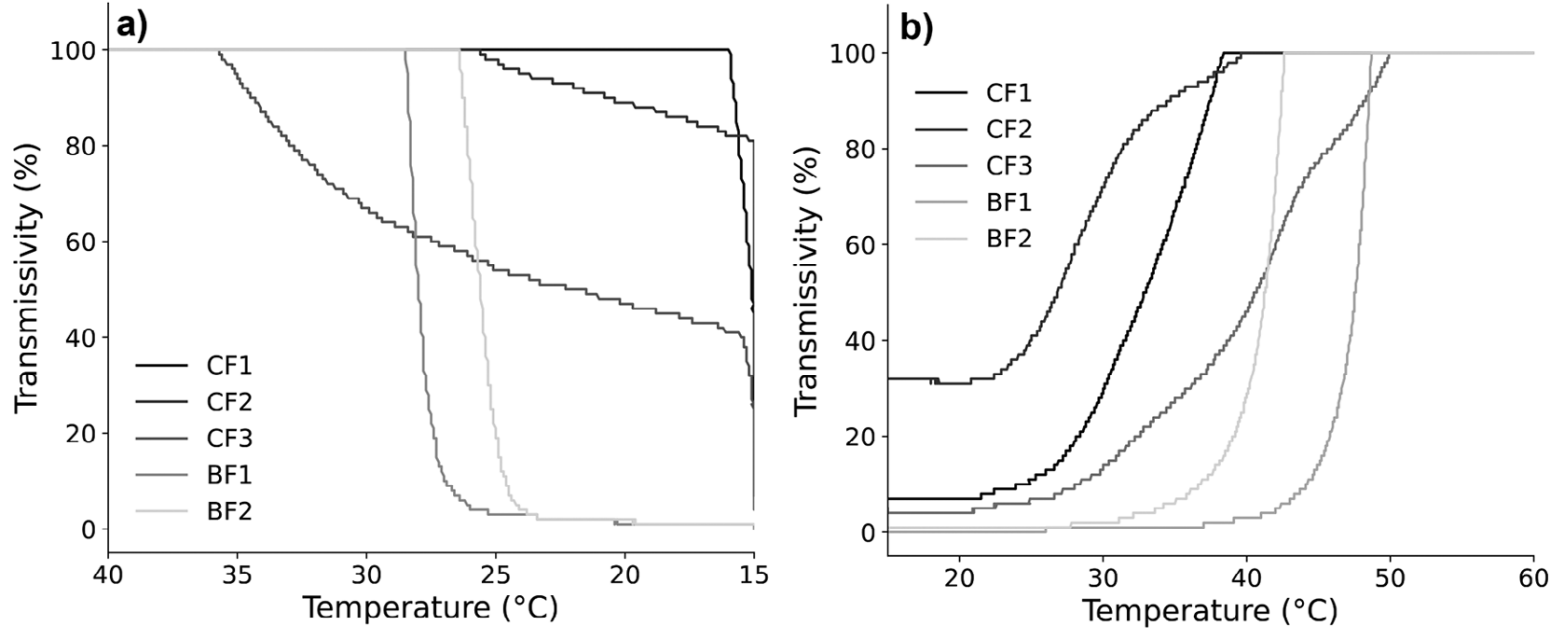
Turbidity profiles during (a) crystallization and (b) melting, approximating the percentage of crystallized fat.

Turbidimetry (Figure 9a,b) was used to follow crystallization and melting for all samples, enabling rapid estimation of melting and crystallization temperatures and general SFC trends. Despite the fact that this technique does not provide a deep structural or a direct thermal characterization it is still a simple and straightforward way to investigate the general crystallization trend of fat systems.

**FIGURE 10:**
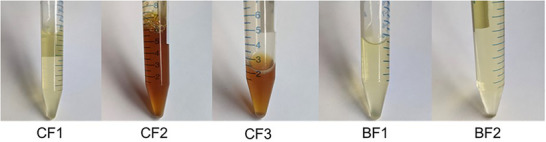
Appearance of the melted investigated samples.

It is worth mentioning that a value of 0% of transmissivity is not necessarily related to the end of the crystallization but may instead be related to the detector saturation [37]. On the other hand, a fully melted sample may not reach 100% and this is particularly evident when looking at the results for colored samples, such as CF2 and CF3 (Figure 10).

Aside from CF2 and CF3, whose transmissivity is unreliable due to their darker color, the more transparent samples show a melting profile in accordance with the SFC curves. Both melting and crystallization curves can be explained by the composition: the highest‐melting BF1 has the highest content in saturated TAGs and FAs, followed by BF2 and finally by CF1. Recorded crystallization and melting temperatures are reported in Table 6.

**TABLE 6:** Melting and crystallization temperatures for all samples measured with turbidimetry.

Sample	Crystallization (°C)	Melting (°C)
CF1	18	35.4
CF2	19.9	31.1
CF3	35	37.5
BF1	29	44.3
BF2	27	43

## 4 Results Summary

Different animal tissue fats and their blends have been analyzed with different and complementary techniques. It has been pointed out how differences in TAG composition drive the melting profile and the crystallization and polymorphic transition kinetics.

Chicken fat samples show a higher fraction of tri‐unsaturated TAGs (and higher PUFA content) compared to beef fat samples, while beef fat samples are richer in mono‐unsaturated TAGs and saturated FAs. Blending with palm fat (CF3) or oleic acid (BF2) changes these distributions and, consequently, modifies the overall crystallization and melting behavior.

These compositional differences had a direct effect on thermal properties: chicken fat samples show lower SFC values compared to beef fat samples. Adding 10% palm fat to CF2 (CF3) slightly increases SFC between 10°C and 40°C, due to higher saturated TAG content, while adding 10% oleic acid to BF1 (BF2) decreases SFC more clearly, especially at lower temperatures.

Synchrotron SAXS/WAXS allows for the investigation of the polymorphic behavior and detailed crystallization kinetics. All samples at the equilibrium showed that the β’ polymorph was the predominant one. Samples with a higher content of saturated TAGs and/or symmetric monounsaturated TAGs showed the presence of β polymorphs as well.

Similarly, static time‐resolved experiments show the predominance of the β’ phase and the presence of β polymorphs in samples with higher saturated TAG content. For higher‐melting samples, the formation of the α polymorph, which was then converted to a more stable polymorph, was detected as well.

The effect of shear on crystallization behavior was also investigated, showing the effect of externally applied forces on the crystallization behavior. For all samples, the direct formation of the β’ polymorph is reported, the formation of β, is instead delayed and it is not detected during the experiment.

DSC provides complementary thermal behaviors that are consistent with the SAXS/WAXS results. DSC attributions were made using peak order/relative stability of polymorphs, cross‐checking with the and the structural results from Section 3.3. This again matched the TAG compositions, showing higher melting phases in CF1, BF1, and BF2.

These trends are confirmed by turbidimetry measurements, showing different melting/crystallization behaviors among samples that matched the SFC measurements. Transmissivity detection was also less reliable on colored samples like CF2 and CF3.

## 5 Conclusions

Reformulating fat‐based products is not a trivial task: linking composition, polymorphic and phase behavior with product properties remains challenging. The growing demand for plant‐based food alternatives further increase the need for a detailed understanding of fat crystallization, so that animal fat alternatives can be effectively designed in order to preserve properties and functionality.

In order to design a plant‐based alternative, it is still necessary to investigate deeply the crystallization behavior of animal fats, which are not as well characterized as other common fat ingredients such as palm oil or cocoa butter.

Among the several techniques that were used to investigate the crystallization behavior, x‐ray scattering was proved to be the most informative one, thanks to the structural information provided, in particular the detection of polymorphs and coexisting phases, even the less abundant ones.

Thanks to different synchrotron x‐ray scattering setups, it was possible to compare the effect of different crystallization conditions on polymorphic and phase behavior. This work showed how shear influences the crystallization behavior, promoting the direct formation of *β*′ phases from the melt while also delaying the formation of *β* crystals, compared with capillary experiments. Interestingly, chicken fat samples with a similar SFC profile showed different phase behaviors, which can be explained by different relative compositions, in particular, by different concentrations of tri‐saturated and symmetric monounsaturated TAGs. Beef fat samples, had higher SFC at lower temperatures but showed similarity in phase and polymorphic behavior to the lower melting chicken fat sample CF1. Moreover, the effect of blending fats with fatty acids and plant‐based fats was investigated as well, showing, respectively, a decrease and an increase in SFC content and change in crystallization kinetics, and different phase behavior. Complementary benchtop techniques such as DSC and turbidimetry matched the results obtained by X‐ray experiments, although for a clear structural characterization, x‐Ray remains a fundamental technique.

Overall, these results provide a structural basis to rationally tune formulation and processing (cooling and shear) to target desired crystallization pathways and thus functional properties in fat‐based food applications.

## Conflicts of Interest

The authors declare no conflicts of interest.

## Data Availability

The data that support the findings of this study are available from the corresponding author upon reasonable request.
